# Parents’ experiences of caring for a young child with type 1 diabetes: a systematic review and synthesis of qualitative evidence

**DOI:** 10.1186/s12887-021-02569-4

**Published:** 2021-04-04

**Authors:** B. Kimbell, J. Lawton, C. Boughton, R. Hovorka, D. Rankin

**Affiliations:** 1 Centre for Population Health Sciences, Usher Institute, University of Edinburgh, Edinburgh, UK; 2 Wellcome Trust - MRC Medical Research Institute of Metabolic Science, University of Cambridge, Cambridge, UK; 3 Department of Paediatrics, University of Cambridge, Cambridge, UK

**Keywords:** Type 1 diabetes, Parents, Child, Qualitative synthesis

## Abstract

**Aims:**

To synthesise the qualitative evidence on parents’ experiences of caring for a child aged ≤8 years with type 1 diabetes to identify: the challenges they encounter; their views about support received; ways in which support could be improved; and, directions for future research.

**Methods:**

We searched Medline, EMBASE, CINAHL, PsycINFO and Web of Science databases to identify qualitative studies reporting parents’ views and experiences of caring for a child with type 1 diabetes aged ≤8 years. Key analytical themes were identified using thematic synthesis.

**Results:**

Fourteen studies were included. The synthesis resulted in the generation of two overarching themes. *Monopolisation of life* describes the all-encompassing impact diabetes could have on parents due to the constant worry they experienced and the perceived need for vigilance. It describes how parents’ caring responsibilities could affect their wellbeing, relationships and finances, and how a lack of trusted sources of childcare and a desire to enable a ‘normal’ childhood constrained personal choices and activities. However, use of diabetes technologies could lessen some of these burdens. *Experiences of professional and informal support* describes how encounters with healthcare professionals, while generally perceived as helpful, could lead to frustration and anxiety, and how connecting with other parents caring for a child with type 1 diabetes provided valued emotional and practical support.

**Conclusions:**

This synthesis outlines the challenges parents encounter, their views about support received and ways in which support might be improved. It also highlights significant limitations in the current literature and points to important areas for future research, including how sociodemographic factors and use of newer diabetes technologies influence parents’ diabetes management practices and experiences.

PROSPERO: https://www.crd.york.ac.uk/prospero/display_record.php?ID=CRD42019128710

**Supplementary Information:**

The online version contains supplementary material available at 10.1186/s12887-021-02569-4.

## Background

Type 1 diabetes is one of the most common chronic childhood conditions and its incidence is rising worldwide [1], including among pre-school aged children [2]. This condition is now mostly managed using flexible intensive insulin regimens, which involve multiple daily tasks (e.g. regular blood glucose monitoring, carbohydrate counting, calculating and administering insulin) and may present different issues and challenges to conventional regimens based on fixed schedules and insulin doses. For young children (those aged ≤8 years), however, most of these tasks are too complex to undertake independently; hence, parents/caregivers typically take on and/or oversee these responsibilities [3]. The physiological, cognitive, behavioural and socio-emotional issues at this developmental stage make diabetes management challenging [4] and clinically recommended blood glucose targets difficult to achieve [5]. Hence, caring for a young child with diabetes can be overwhelming and stressful for parents, and can affect wider family life [6].

Qualitative studies have explored parents’ experiences of caring for a child with type 1 diabetes in a range of contexts and situations, such as following diagnosis, using different diabetes management regimens and whilst managing transitions [7–11]. Synthesising bodies of qualitative literature can help clarify understanding of a phenomenon, identify gaps and ambiguities in the existing literature, and inform decision-making by policymakers and healthcare practitioners [12]. However, syntheses of qualitative or mixed-methods research involving parents of young children with type 1 diabetes remain scarce and have generally focused on specific aspects of their experience, such as their psychological reactions to their child’s diagnosis [13] or their use of diabetes technologies [14]. To date, no reviews have focused on parents’ *everyday* experiences of caring for a young child with type 1 diabetes. This review aims to address this gap. By identifying, examining and synthesising the qualitative evidence on parents’ experiences of caring for a young child with type 1 diabetes, we sought to: (1) describe the published evidence base; (2) identify the challenges parents encounter when managing their child’s diabetes; (3) explore their views about support received from health professionals and other sources; (4) identify ways in which support could be improved; and (5) identify gaps in the evidence base and directions for future research.

## Methods

We followed Thomas and Harden’s thematic synthesis approach, which is well suited to reviews focused on individuals’ perspectives and experiences [15]. This approach involves a systematic search of relevant literature, quality appraisal of the included studies and three distinct stages of data manipulation: (1) line-by-line coding, (2) organising codes into descriptive themes, and (3) developing analytic themes. Our reporting follows the guidelines for Enhancing Transparency of Reporting the Synthesis of Qualitative Research (ENTREQ) and Preferred Reporting Items for Systematic Reviews and Meta-Analyses (PRISMA) [16, 17].

Details of the protocol for this systematic review and synthesis were registered on PROSPERO (https://www.crd.york.ac.uk/prospero/display_record.php? ID=CRD42019128710).

### Search strategy

We identified papers for inclusion from a systematic search of electronic databases (Medline, EMBASE, CINAHL, PsycINFO and Web of Science). Working with a medical library science professional, we developed a search strategy that drew on existing literature and a combination of Medical Subject Heading (MeSH) terms and keywords relating to our target condition, population and methodology. Our searches were also informed by the SPIDER (Sample, Phenomenon of Interest, Design, Evaluation, Research type) approach to identifying qualitative literature [18] and search terms were tailored to suit each database. We screened the reference lists of included studies and relevant reviews identified by the search to identify further papers for inclusion. We limited our search to papers published from 2002 onwards, as this was the time when flexible intensive insulin regimens began to be widely used as part of routine clinical care [19]. A sample search strategy for Medline database is presented in supplementary figure Fig. S1.

### Study selection and screening

Our choice of age cut-off at ≤8 years was informed by the literature highlighting the high level of parental responsibility for diabetes management tasks in this younger age group [3]. Subsequently, increasing maturity and independence sees children assume progressively more responsibility for their own diabetes care; this transition changes parents’ role in their child’s diabetes management, and thus their experiences, and was outside the scope of this review.

We included peer-reviewed papers published in English if they reported: (1) primary research using qualitative methods or mixed-methods studies reporting qualitative data separately; and (2) views and/or experiences of parents and/or caregivers of children with type 1 diabetes ≤8 years of age. This included studies which also reported the views of parents of older children, but where findings pertaining to those with children aged ≤8 years and cutting across age ranges were clearly discernible. We had originally excluded some cross-cutting papers involving only a small number of parents of children in our target age range. However, a later re-evaluation found these studies contributing important cultural and sociodemographic dimensions to the overall analysis, which warranted their inclusion. We excluded papers if they reported: (1) non-primary research; (2) only quantitative research; (3) data that focused exclusively on: parent/caregiver views and/or experiences regarding their child being diagnosed or immediately after diagnosis (which have been reviewed elsewhere [20]); parents of children older than 8 years with type 1 diabetes; and, adults with type 1 diabetes reporting their own experiences of living with type 1 diabetes.

Search outputs were imported into EndNote X8, then exported, de-duplicated and screened using Covidence systematic review management software (Veritas Health Innovation, Melbourne, Australia). To reduce selection bias, two authors (BK and DR) independently screened the titles and abstracts of identified records and compared and agreed their selections. Full texts were retrieved for any papers that appeared to meet the eligibility criteria. Disagreements on the final selection were minimal and resolved through discussion without need for third-party arbitration.

### Data extraction and quality assessment

BK extracted the following data from the included studies: author(s); year of publication; country; study aims; sample size; parent and child characteristics; methodology. For each paper, we imported full ‘Results’ and ‘Discussion’ sections into NVivo 10 (QRS International, Doncaster, Australia). We extracted quotations and descriptive reporting of parents’ accounts from Results sections only when this material could be clearly attributed to parents of children ≤8 years of age. No findings (quotations or descriptive material) were extracted which reported the views of parents of children aged > 8 years. In keeping with our aim to identify ways in which support for parents could be improved, we extracted recommendations in Discussion sections proffered by the primary authors. Recommendations made by primary authors were only extracted when these could be clearly attributed to children ≤8 years of age, or where these were cross-cutting.

BK and DR evaluated each study using the CASP (Critical Appraisal Skills Programme) quality appraisal tool for qualitative studies [21]. This tool consists of 10 questions considering different aspects of study validity and the perceived value of each study’s contribution. The purpose of this systematic appraisal process was not to exclude studies, but to consider strengths and limitations of the included studies.

### Data analysis and synthesis

We conducted a 3-stage thematic synthesis informed by Thomas and Harden’s thematic synthesis approach [15]. First, findings from included articles relevant to the aims of the review were coded using free codes that remained close to the original meaning in the primary studies. Second, we compared similarities and differences between the free codes before grouping related data segments into descriptive themes. Finally, we considered the patterns and relationships between these themes to develop interpretations beyond the primary data and generate overarching analytical themes. We then used the same process to compare recommendations made in the Discussion sections of selected articles, by comparing similarities and differences to develop descriptive themes, followed by the generation of analytical themes. We ensured that data pertaining to findings and recommendations were kept separate. This was done to distinguish between themes arising directly from participants’ data (findings) and the thematic synthesis of recommendations developed by authors in response to their findings. BK independently coded the extracted data and undertook the synthesis. To reduce bias and enhance rigour, the resultant outputs were discussed with two other review authors (DR and JL) to consider any additions or changes and agree on the final analytic themes.

## Results

**Fig. 1:**
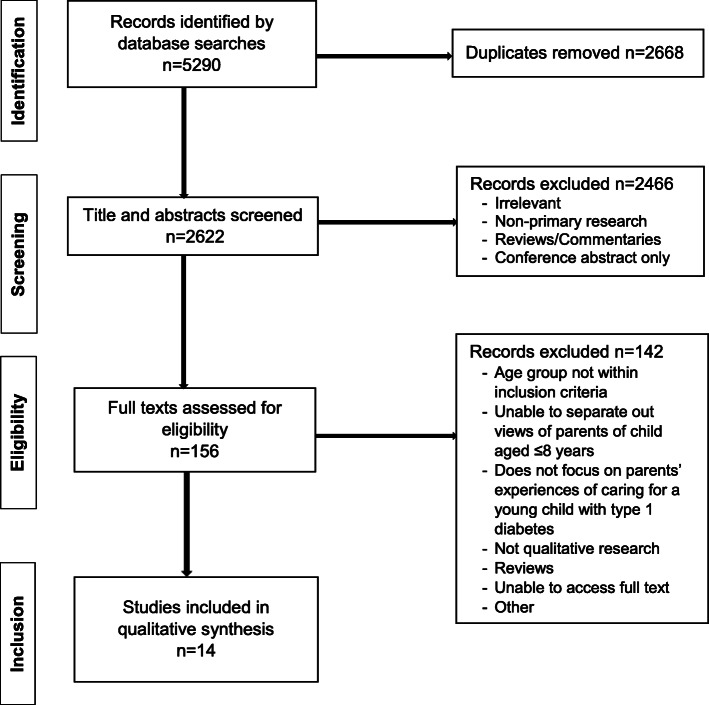
PRISMA flow chart of study selection process

The search identified 2622 unique records (see Fig. 1). Of these, 2466 papers were excluded after titles and abstracts were screened for relevance. Full-text review of the remaining 156 studies led to the exclusion of 142 papers that did not meet eligibility criteria. Screening of reference lists of included studies and relevant reviews identified by the search did not identify further papers for inclusion. This resulted in 14 studies being included in the synthesis.

### Study characteristics

The 14 included papers reported the views and experiences of 274 parents in seven countries: Canada [22], United States [23–27], United Kingdom [28–30], Sweden [31, 32], Norway [33], Iran [34] and Palestine [35]. Four papers reported exclusively on the experiences of parents of children aged ≤8 years (*n* = 80) [23, 25, 26, 33]. The remaining 10 also included parents of older children and provided insufficient detail to determine the number of parents with children in our target age group. The provision of information about study participants’ sociodemographic characteristics varied greatly. Across all studies, the majority of parents were reported as being: married or co-habiting, qualified to higher education level and in employment. Approximately half of papers specified participants’ ethnicity and reported this as mostly, or exclusively, white/Caucasian [23–27, 29]. All studies employed interviews; one additionally used online focus group discussions. Most studies considered parents’ holistic experiences of caring for a young child with type 1 diabetes, with some focusing specifically on the experiences of mothers [23, 32, 34] and fathers [25, 31], respectively. Two papers described parents’ everyday experiences of managing their child’s condition using insulin pumps [24, 29]. Table 1 outlines the key characteristics of the included studies.

**Table 1:** Characteristics of studies included in the qualitative synthesis

Reference	Country	Study aim	Parent characteristics	Child characteristics	Method	Data analysis
Boman et al., 2013 [31]	Sweden	To explore and discuss how fathers involved in caring for a child with T1D experience support from paediatric diabetes teams in everyday life.	*n* = 11 (all fathers) Age: 37–51 years Cohabiting with mother: *n* = 7 Higher education: *n* = 5	*n* = 11 (≤8yo *n* = 6) Age: 4–16 years Diabetes duration: 2–8 years	Online focus group discussion (*n* = 6 fathers); semi-structured interviews (*n* = 8 fathers) (mix of phone and face-to-face); both (*n* = 3 fathers)	Constructivist grounded theory analysis
Elissa et al., 2017 [35]	Palestine	To explore the experiences of daily life in children with T1D and their parents living in the West Bank in Palestine	*n* = 10 (6 mothers) Age mothers: 28–49 years Age fathers: 32–42 years Cohabiting: all Higher education: *n* = 3 In employment: *n* = 4 (fathers) Rural or camp living: *n* = 4	*n* = 10 Age: 8–16 years Diabetes duration: < 5 years (*n* = 3); 1–5 years (*n* = 4); > 5 years (*n* = 3)	Face-to-face interviews	Qualitative content analysis as per Graneheim & Lundman (2004)
Iversen et al., 2018 [33]	Norway	To explore the lived experience of being mothers and fathers of a young child with T1D aged 1–7 who had had the diagnosis for at least 1 year.	*n* = 15 (8 mothers) Age mothers: 26–40 years (m = 30) Age fathers: 29–46 (m = 38) Cohabiting: 7 couples, 1 single mother In employment: all	*n* = 8 Age: 1–7 years Age at diagnosis: 1–5 years Diabetes duration: 1–6 years MDI (pen): *n* = 1 CSII: *n* = 7	In-depth face-to-face interviews (one by telephone)	Interpretative phenomenological methodology as described by Van Manen
Khandan et al., 2018 [34]	Iran	To explore the experiences of mothers with diabetic children after the transfer of caring role	*n* = 11 (all mothers) Age: 30–48 years Cohabiting: *n* = 9 Higher education: *n* = 8 In employment: *n* = 5	*n* = 11 Age: 7–14 years (≤8yo *n* = 3) Diabetes duration: 12–96 months	Semi-structured and open-ended face-to-face interviews	Analysis as per Colaizzi
Lawton et al., 2015 [28]	UK	To explore the difficulties parents encounter in trying to achieve clinically recommended blood glucose levels.	*n* = 54 (38 mothers) Age all parents: 25–51 years (m = 40.6 ± 6.1) Cohabiting: 70% Higher education: 27.8% In employment: 68.5%	*n* = 41 Age: 2–12 years (m = 8.4 ± 2.5) Age at diagnosis: 3–10 years (m = 5.2 ± 2.1) Diabetes duration: 1–11 years (m = 4.1 ± 2.9) CSII: 31.7%	In-depth face-to-face interviews	General theoretical and procedural direction taken from Grounded Theory research
Lindström et al., 2017 [32]	Sweden	To experience how mothers experiencing burnout describe mothering a child with diabetes, with special focus on their need for control and self-esteem.	*n* = 21 (all mothers) Age: 31–50 years (m = 41) Cohabiting: 85.7% Higher education: 71.5% In employment: 90.4%	*n* = 22 Age: 3–17 years (m = 10.7) Diabetes duration: 1.5–15 years (m = 5.3) CSII: 77%	Semi-structured, face-to-face interviews	Inductive content analysis
Marshall et al., 2009 [30]	UK	To explore and describe the experiences of children and their parents living with T1D from diagnosis onwards	*n* = 11 (10 mothers) Ethnicity: Asian, Eastern European, Jamaican, Irish, English backgrounds	*n* = 10 (≤8yo *n* = 4) Age: 4–17 years Diabetes duration: 10 months – 8 years	Conversational interviews	Van Manen’s phenomenological approach to thematic coding
Patton et al., 2016 [26]	US	To describe parents’ perceptions of healthful eating for T1D in families of young children and identify factors related to parents’ dietary management in young children.	*n* = 23 (21 mothers) Age all parents: 27–49 years (m = 35.7 ± 5.1) Married: 83% Higher education: 87%	*n* = not specified Age: 2–6.9 years (m = 4.6 ± 1.3) Ethnicity: 78% non-hispanic white, 13% hispanic, 9% black Diabetes duration: m = 2.0 ± 1.5 years CSII: 87%	Semi-structured, face-to-face interviews	Guided by a grounded theory approach
Perez et al., 2018 [27]	US	To explore how parents negotiate the uncertainty surrounding T1D	*n* = 29 (mother/father not specified) Age all parents: 33–50 (m = 44) Ethnicity: all Caucasian/white Married: *n* = 28 (97%) In full-time employment: *n* = 18 (stay-at-home: *n* = 11) Most identified household income as middle to upper-middle class	*n* = 30 Age: 2–17 years (m = 10.9) Age at diagnosis: 13 months - 13 years (m = 6.5 years) Diabetes duration: 4 months - 10 years (m = 4.39)	Interviews (by phone *n* = 26)	Thematic analysis as per Braun & Clarke (2006)
Rankin et al., 2015 [29]	UK	To explore parents’ experiences of using an insulin pump to manage their child’s diabetes, including their views about the benefits and challenges for themselves and their child.	*n* = 19 (13 mothers) Age all parents: 34–44 years (m = 40.1 ± 3.7) Ethnicity: all white British Married or cohabiting: *n* = 18 Higher education: *n* = 9 In employment: *n* = 12	*n* = 14 Age: 3–12 years (m = 8.4 ± 2.8) Age at diagnosis: 1–6 years (m = 3.8 ± 2.1) Length of time on pump: 1–4 years (m = 2.2 ± 1.2)	Face-to-face interviews	Thematic analysis using the method of constant comparison
Sullivan-Bolyai et al., 2003 [23]	US	To provide a detailed description of day-to-day management experiences of mothers raising young children under 4 years with T1D.	*n* = 28 (all mothers) Age: m = 33 ± 5.24 years Ethnicity: 89% white Married: 86% Education: m = 15 ± 2.5 years Not working outside of home: *n* = 15	*n* = 28 Age: m = 2.9 ± 0.6 years Diabetes duration: m = 1.25 ± 0.7 years	Face-to-face interviews	Naturalistic inquiry
Sullivan-Bolyai et al., 2004 [24]	US	To describe the experiences of parents managing the T1D of their young children using an insulin pump.	*n* = 21 (14 mothers) Age all parents: m = 38 ± 3 years Ethnicity: all Caucasian Married: *n* = 20 Education: m = 16 ± 2 years	*n* = 16 Age: 2–11 years (m = 7 ± 2 years Length of time on pump: 3–36 months (m = 16 ± 11)	In-depth, face-to-face interviews	Qualitative content analysis as described by Sandelowski
Sullivan-Bolyai et al., 2006 [25]	US	To describe fathers’ experiences in parenting and managing the care of their young children’s day-to-day diabetes regimen.	*n* = 14 (all fathers) Age: m = 36 ± 2 years Ethnicity: all white Married: all Education: m = 16 ± 2 years In employment: all	*n* = 15 Age: 2–8 years (m = 5 ± 2) Diabetes duration: 2 weeks – 3 years (m = 1.4 ± 0.8 months)	Face-to-face interviews	Qualitative content analysis
Watt, 2017 [22]	Canada	To explore the emotion work of doing worry that parents engage in when caring for their children with diabetes.	*n* = 7 (5 mothers) Age all parents: 34–53 years (m = 44) All 2-parent, middle class families Education: all higher education	*n* = not specified Age: 18 years or younger Age at diagnosis: 9 months – 14 years	In-depth interviews in the context of institutional ethnography (not clear if face-to-face or phone)	Analysis guided by Smith’s (2005) conception of work and analytic questions suggested by IE scholars (McCoy, 2006)

### Quality assessment

Using the CASP quality appraisal checklist [21], we concluded that all 14 studies had clearly justified and stated research aims, appropriately employed qualitative methodology and provided sufficient information about their data collection processes. However, in some cases it was difficult to determine the rigour of data analysis from the limited information provided. Furthermore, several papers lacked detail regarding their consideration and mitigation of potential researcher influence and ethical issues. In respect of their wider contribution, we rated 10 of the 14 studies as being of good value, three of medium and one of low value. The study rated low value used mixed methods to report on a narrow topic area (parents’ perceptions of healthy eating for children with type 1 diabetes) [26]. See supplementary Table S1 for the completed CASP scoresheet.

### Synthesis findings

Below, we present two overarching analytical themes resulting from our synthesis, *Monopolisation of life* and *Experiences of professional and informal support*, with each theme comprising several subthemes. Primary authors’ recommendations for how parent/caregiver support could be improved in respect of the issues identified are summarised in Table 2.

**Table 2:** Primary authors’ recommendations to improve parent/caregiver support in relation to each analytical theme

**1. Monopolisation of life**	
To help reduce the detrimental impact on parents’ psychological and emotional wellbeing, the primary authors recommended that healthcare professionals could: ascertain and address issues related to hypoglycaemia concerns, lapses in confidence and sleep [23]; and, provide encouragement and support by acknowledging the unpredictability of diabetes and treatment outcomes [22]. More general recommendations included professionals needing to familiarise themselves with the symptoms of burnout [32], and helping parents address any harmful emotions related to their caregiving situation [33]. This could involve: teaching parents strategies to manage negative feelings about the child being ‘different’ because of diabetes [26]; and, assessing and encouraging parental self-care, including helping to identify sources of respite [23]. Finally, to help reduce anxieties related to social stigma and gendered impacts, primary authors recommended that healthcare professionals seek to improve public awareness and understanding of type 1 diabetes [27, 34, 35]. To help relieve the care burden on mothers and encourage fathers’ involvement, primary authors recommended that professionals should, from the outset, set the expectation that (where possible) both parents attend clinic consultations [28] and that, for respite and emergency purposes, both should be involved in their child’s diabetes management [25]. This recommendation could be supported by working with parents to develop a ‘division of labour’ plan [23]. To alleviate parents’ concerns regarding potentially inappropriate diabetes management in daycare settings (e.g. nurseries, schools, playgroups), primary authors recommended that healthcare professionals should help educate staff on safe management practices [23] and, where possible, broaden their outreach work in these settings to increase the number of people available to support the child’s diabetes management [28]. To address potential financial pressures related to the child’s diabetes treatment, primary authors recommended that healthcare professionals should provide parents with financial guidance about all aspects of diabetes management [27] and offer referral to charitable organisations where appropriate [34].	
**2. Parents’ experiences of professional and informal support**	
To address parents’ concerns regarding their diabetes management education and avoid mixed messages, primary authors recommended that healthcare professionals should develop and follow an agreed-upon teaching plan; this should include the option of booster sessions, which revisit information and techniques taught at the time of diagnosis [25] and take into consideration individuals’ differing speeds of learning and developing confidence [24]. To alleviate potential tensions between parents’ and professionals’ views regarding diabetes management, primary authors recommended that healthcare professionals should educate parents on their specific clinical perspective [28], while also using parents’ knowledge regarding their unique family situation and the child’s individual needs to inform treatment decisions [28, 31].

1. ***Monopolisation of life***

#### Impact on physical, psychological and emotional wellbeing

Across the studies, parents noted how the complexities and unpredictability of type 1 diabetes made it a ‘very tiring disease’ to manage [25]. They described living in a perpetual state of watchfulness [22, 23, 33] and physical and mental readiness to take action, because ‘[T] hings may change in minutes’ [33]. Consequently, their child’s diabetes was permanently present in their minds. As one father explained, ‘even if you are not thinking about it [the illness], you are’ [25].

Hypoglycaemia, in particular, was an all-pervasive concern [22, 23, 28] borne from several considerations: the child being too young to recognise and report low blood glucose (‘he doesn’t have a clue, because he’s just learning to talk’ [28]), some children’s poor hypoglycaemia awareness [28, 29]; and, parents’ awareness of the potential deadly consequences of hypoglycaemic events [22, 28, 32]. Parents’ concerns were greatest during the night, when they worried that severe hypoglycaemia might go undetected and threaten their child’s safety. To alleviate their fears, they described testing blood glucose regularly throughout the night, leading to exhaustion and chronic sleep deprivation [22, 23, 33]. Some parents recognised that their actions could be borne from irrational fears, but preferred being hyper-vigilant to having potential future regrets [22, 29]. Some, like this mother, also acknowledged that their efforts came at a cost to their own health and wellbeing:

‘I am satisfied in one sense since NN is feeling fine. At the same time, I feel unhappy when I think about not sleeping, feeling anxious and feeling tired and moody all the time.’ [32]

Furthermore, despite their child’s young age, many parents already worried about how diabetes would affect his/her life in the future [27, 30, 34, 35]:

‘I am always thinking about his future. I wonder what will happen to his body. Can he be successful in his life? I do not know; the future is unclear.’ [34]

These concerns could be influenced by sociocultural norms and expectations. Parents of young girls in the studies conducted in Iran [34] and Palestine [35] described worrying about their daughter’s diabetes harming her chances in marriage, lest she be viewed as less desirable and at risk of passing the condition on to her own children.

In light of parents’ varied and constant concerns, many worried about how their emotions might be perceived by, and affect, the child, because, as this mother explained, ‘it’s hard not to transfer that worry onto him all the time. I know I don’t want him to feel worry not going to places or doing things or that sort of thing’ [22]. Consequently, they described deliberate efforts to hide their fears, worries and exhaustion by adopting an ‘outward façade’ [22, 32]. Additionally, some mothers reported depression, weight problems, migraines and episodes of hospitalisation, which they linked to the burden of their caring responsibilities [23].

#### Impact on relationships

Several studies highlighted how caregiving responsibilities not only monopolised parents’ own lives, but also affected their relationship with the child [23, 30, 32, 33]. Some parents observed how diabetes had ‘come between me and my child, and to me that was kind of a feeling of loss’ [33]. Managing their child’s condition was described as requiring an atypical level of caregiving input [32], with some mothers likening their experience to caring for a newborn [23].

Mothers and fathers also described how their relationships with one another had changed as a result of having to ‘live with constant attention directed at the diabetes condition’ [33]. Mothers typically shouldered the main caring responsibilities [23, 25, 32], with fathers being more willing to be involved in diabetes care when it involved technology [24]. However, fathers still played an important role, especially by providing emotional support and respite to mothers [23, 25, 32, 34]. Some studies indicated potential gender differences in parents’ attitudes and approaches to their child’s diabetes management, with fathers being more relaxed than mothers in this regard [24, 25, 32]. This could sometimes lead to conflict between parents, but also encouraged more in-depth communication about how best to manage their child’s diabetes [24].

#### Impact on personal choices and activities

Parents described caring for a child with type 1 diabetes as a full-time job [32, 33]. They noted that the unpredictability of the condition required them to constantly plan ahead [33]. Accommodating regular clinic appointments required time and flexible employment [25]. Having their child looked after in a daycare facility, including nursery or school, did not necessarily provide respite. Indeed, it could create additional work, as parents needed to ensure that staff were educated about their child’s specific care needs and make themselves available throughout the day to answer questions or attend the facility as required [33]. Moreover, the unpredictability of their child’s eating and physical activity while at school/nursery could add to parents’ anxieties about their child’s safety [28]. Mothers also reported feeling concerned about staff’s (in) ability to provide appropriate diabetes care and some chose not to place their child in daycare for that reason [23]. Similarly, many parents felt unable to entrust the care of their child to relatives and others in the community, as they perceived them as largely ignorant about diabetes and/or insufficiently vigilant about its management [22, 24, 28, 32]. Consequently, many mothers curbed personal activities to be available to care for their child [23, 28, 35]. As one mother explained:

‘I didn’t go to many places, because she couldn’t be with me and no one else can take care of her but me.’ [35]

These concerns could also affect mothers’ employment decisions, with some quitting work or reducing their working hours to allow them to care for their child at home [28]. However, others described how, despite wanting to be stay-at-home caregivers, they needed paid employment to afford their child’s diabetes treatment costs [34]. Importantly, this financial strain related to their child’s diabetes care was also reported by parents who self-identified as middle- to upper-middle class [27] and were in possession of medical insurance, as this did not always cover all necessary expenses [34, 35].

Finally, several studies described how parents were determined not to let diabetes dominate their child’s life [22, 25, 26, 28, 30, 32, 33], so that the child could ‘have her innocence, to go out and play and feel like a normal child without feeling there is something different with her’ [28]. To facilitate this ‘normality’, parents adopted strategies that required even more of their time and effort, such as becoming actively involved in school and social activities (e.g. their child’s sports team) to allow the child to participate while ensuring a watchful eye on their glucose needs [25] and temporarily relaxing the child’s food regimen and later correcting high blood glucose if necessary [26, 28]. Caring for a young child with diabetes also had an all-encompassing impact on wider family life. Some parents reported modifying their own and/or their family’s eating practices to make managing mealtimes easier [26]. Bedtimes, leisure activities and holidays were also often adapted to accommodate the child’s needs and limited opportunities for spontaneity [32].

#### Diabetes technologies: lessening the impact

While most studies illustrated the pervasive impact of the child’s diabetes on parents’ lives, two studies highlighted how using an insulin pump could alleviate some of the stresses and constraints they experienced [24, 29]. Although parents reported needing to undertake some additional tasks, such as dealing with occasional mechanical problems [24] or more frequent blood glucose checking [29], they also described how pump use had helped reduce the ‘slavery of diabetes management’ [24] because they no longer needed to administer basal insulin at specific times of day [29], could approach eating and snacking more flexibly due to the ease with which bolus doses could be administered via the pump [24, 29], felt less fearful about their child being cared for by others [24, 29] and found others more willing to babysit [24]. Parents also reported finding it easier to achieve good blood glucose control using a pump due to the ability to administer smaller (more precise) insulin doses, having fewer variables (e.g. only one type of insulin) to manipulate to manage glucose excursions and the pump’s data log and bolus advisor helping to reduce management errors [24, 29]. Finally, parents in another study described how using a continuous glucose monitor had helped make treatment decisions easier as it gave them convenient access (via a smart phone app or digital platform such as Nightscout) to real-time blood glucose information and allowed them to review how their child’s body responded to different insulin doses throughout the day [27].

2. ***Parents’ experiences of professional and informal support***

#### Experiences of professional support

Parents received their initial education about diabetes management from hospital paediatric diabetes teams. However, they described how sometimes ‘one nurse would come in and say do it this way, another would come in and show us a different way’ [25], resulting in inconsistencies in the information received. Moreover, parents across several studies considered their initial training inadequate preparation for the daily challenges of caring for a young child with type 1 diabetes [23, 25–27, 34]. As this father noted:

‘It is like being handed a big city phone book and you have to learn all the names before you go home.’ [25]

While diabetes teams were generally considered a helpful resource, some parents felt that professionals did not always appreciate the complex and dynamic nature of managing diabetes at home [31] and the considerable effort this required [23]. Furthermore, staff not making time to answer questions or calls, avoiding discussion of more holistic issues and offering inaccurate or inconsistent advice could undermine parents’ trust in their diabetes team [31]. Some parents described how they felt stressed and anxious in the run-up to clinic appointments for fear of being reprimanded for a (perceived) lack of effort and not meeting blood glucose targets [23, 28, 32]. This fear also led to some actively withholding information from the diabetes team [23] and was felt even in the absence of any critical comments from staff [32].

Several studies also highlighted potential conflict between parents’ and professionals’ diabetes expertise. The fathers in Boman’s study described a mismatch between their own personal experiences of caring for a child with diabetes and the general recommendations and goals put forward by the diabetes team [31]. Parents in another study felt that healthcare professionals had unrealistic expectations of what was achievable in terms of their young child’s blood glucose control [28]. Indeed, many emphasised how their unique personal understanding of their child’s individual needs and their impact on everyday life provided them with insights that extended beyond professionals’ focus on glycaemic control [22, 28, 31]. As this father of a 4-year-old reported:

‘I have a larger backpack than the professionals’ knowledge of HbA1c. Yes, it’s an individual who is affected, but in everyday life it [the diabetes] controls the whole family’s life, and then you have to have more in your backpack than just HbA1c.’ [31]

Relatedly, some parents described how professionals tended to focus exclusively on the needs of the child and failed to acknowledge how some parents may be struggling to cope with the strains of diabetes management in the context of wider family life [32].

#### Experiences of informal support

Parents described drawing on informal sources to support the management of their child’s diabetes. Most often, this involved their spouses/partners [23, 25, 33] or other family members [23], although their support could be limited due to relatives’ poor diabetes knowledge and understanding [32]. While parents craved social contact with other families, their caring responsibilities made them feel different to others and they reported struggling to feel fully present in social situations [33]. Some parents, like this mother, credited support groups with making them feel less isolated and able to vent their frustrations about the challenges of providing diabetes care:

‘I am in a diabetes support group with moms and I find I’ve learned a lot from what other moms do … I can say, oh my goodness, today is making me crazy and I can’t figure it out and diabetes is not fun right now.’ [22]

Moreover, parents considered their peers a vital source of information when professional advice was deemed insufficient [34] or, as this mother explained, difficult to access [27]:

‘Facebook groups were also super helpful, because it was really nice to be able to post a question like, “How do you guys do this, or what should I do about this?” … because we did have the number to call, but getting hold of the doctor or educator was just a huge pain, and sometimes you don’t know if your question is big enough to call the doctor about.’ [27]

## Discussion

This review is the first to synthesise and describe the findings from qualitative studies, which report parents’ everyday experiences of caring for a child aged ≤8 years with type 1 diabetes. It highlights the all-encompassing, relentless and enduring nature of parents’ care experiences and how their lives are dominated by constant worry, the need to be vigilant and a desire to enable their child to have a ‘normal’ childhood. Moreover, the synthesis illustrates how caregiving responsibilities could be detrimental to parents’ own physical, psychological and emotional well-being, relationships, personal choices and everyday activities. Parents’ encounters with healthcare professionals, while generally perceived as helpful, could add to their anxieties and frustrations, as could lack of access to trusted sources of childcare and informal support. Conversely, connecting with other parents who had a child with type 1 diabetes constituted an important source of emotional and practical support. The synthesised recommendations by primary authors presented in Table 2 highlight ways in which clinical practice might be adapted to help alleviate parents’ care burden, improve their emotional and educational support, and foster more collaborative working between parents and professionals.

Some parents, particularly mothers, described how they were forced to make decisions about employment based on their child’s diabetes care needs and associated expenses. Furthermore, even parents who self-identified as middle-class and were in possession of medical insurance reported experiencing diabetes-related financial strains due to at least some treatment supplies needing to be paid for through personal means. Resonating with these findings, a survey conducted with parents of young children with type 1 diabetes found that having a child with diabetes influenced the employment decisions of 60% of parents (89.5% of them mothers), with nearly one quarter reducing or quitting work and others maintaining employment for financial reasons [36]. Research has also shown that caring for a child with type 1 diabetes was significantly more detrimental to their work and finances compared with parents of children with other or no special healthcare needs [37]. The study samples in our synthesis were skewed towards co-habiting and working parents; hence, our findings raise important questions and concerns about how parents living in low-income countries or on low incomes (including single parents, who are more likely to report lower incomes and benefit dependency [38]) manage the practical and financial demands of their child’s diabetes care.

Some parents described how using insulin pumps and glucose sensors helped reduce the stresses and constraints diabetes management placed on everyday life. The use of insulin pumps in paediatric populations has risen considerably in recent years [39] and insulin pump therapy is now the recommended method of insulin administration in young children [40]. These developments suggest that greater numbers of parents are now using insulin pumps than when some of the included studies were conducted. Research suggests that newer technologies, such as continuous glucose monitors and closed-loop systems, are likely to help further ease the burden of diabetes management. For example, use of continuous glucose monitors may lessen parental anxiety due to the device alerting them to hypo- and hyperglycaemia [41], while those able to monitor their child’s glucose data remotely may experience improved sleep and greater lifestyle freedoms [42, 43]. Similarly, while user evaluations of closed-loop systems have mainly involved older participant groups with type 1 diabetes and/or their parents [44–48], preliminary trials involving very young children suggest that this technology can help parents feel less burdened by diabetes management tasks and facilitate better sleep [49].

Several parents reported benefitting from the emotional and practical support provided by other parents of children with type 1 diabetes via support groups and online fora. Conversely, while parents were generally appreciative of the support provided by healthcare professionals, some described how this contact could make them feel frustrated and anxious. They also described receiving inadequate diabetes education following their child’s diagnosis and conflicting messages from different healthcare professionals. These issues are noteworthy as, arguably, they could be adding to the psychological and emotional burden parents experience. Other studies have described how parents wish for a tailored, collaborative approach to their education and clearer, more sensitive communication from diabetes professionals [50]. Moreover, it has been noted that parents feeling anxious during diabetes consultations can affect their ability to concentrate, and thus assimilate, the information provided [51]. As appropriate patient education and communication is critical to achieving positive behaviour change in diabetes management [52], diabetes teams should urgently consider the quality of their communication and parents’ emotional needs during clinical encounters. Primary authors’ recommendations, such as adopting a collaborative approach to engaging with parents (Table 2), provide a useful starting point for diabetes teams to consider and build upon.

This review and synthesis was conducted in accordance with established methods for the systematic reviewing, appraising and synthesising of qualitative studies [15, 21] and reported according to published guidelines [16, 17]. Nevertheless, we acknowledge that syntheses, by their nature, cannot convey the contextual richness of the individual studies upon which they draw. We also recognise that our decision to exclude papers not published in English may have resulted in the final sample containing fewer studies from lower-income countries. However, our reporting is strengthened by the consistency of findings observed across the primary studies, despite these having been conducted in a diversity of countries with different cultures and healthcare systems. We also recognise the potential influence of our unique perspectives as UK-based, non-clinical researchers throughout the analytic process and in the presentation of results.

The limitations inherent in the primary studies included in this synthesis highlight important considerations for future research. The study samples were biased towards parents who were married or co-habiting, qualified to higher education level, in employment and white/Caucasian. Consequently, the experiences and views presented in this synthesis may not reflect those of other parents caring for a young child with type 1 diabetes. Indeed, while we found good consistency of findings across the studies, they did indicate potentially divergent challenges related to income and cultural norms. Other studies suggest that education, financial status, family make-up and ethnicity may differentially affect parents’ ability to manage and cope with diabetes [53, 54] and, importantly, influence children’s diabetes outcomes [55]. Consequently, it is vital that future research considers the experiences and views of parents of different demographic and socioeconomic backgrounds and those living in low-income countries and settings. Moreover, as parents’ experiences, and thus support needs, may be more diverse than the current literature shows, providing more detailed participant data will help practitioners draw more nuanced conclusions from study findings. Finally, given the potential positive impact of newer diabetes technologies, such as closed-loop systems, qualitative studies could explore the experiences of parents caring for very young children with type 1 diabetes using these newer technologies and assess whether, and how, they help address some of the challenges highlighted in this review.

## Conclusions

The current literature consistently describes caring for a young child with type 1 diabetes as an all-encompassing and relentless undertaking, which can have a detrimental impact on parents’ own well-being, relationships, personal choices and everyday activities. However, significant limitations and gaps in this literature mean that parents’ experiences may in fact be more diverse than is currently recognised, which could have implications for the support they require from healthcare professionals. In particular, we recommend that future research should explore how sociodemographic factors and use of newer diabetes technologies influence parents’ diabetes management practices and experiences of caring for a young child with type 1 diabetes.

## Supplementary Information

**Additional file 1: Table S1**. CASP quality appraisal scoresheet

**Additional file 2: Fig. S1**. Exemplar search strategy from Medline database

## Data Availability

Not applicable.

## Abbreviations

ENTREQ: Enhancing Transparency of Reporting the Synthesis of Qualitative Research
PRISMA: Preferred Reporting Items for Systematic Reviews and Meta-Analyses
MeSH: Medical Subject Heading
SPIDER: Sample, Phenomenon of Interest, Design, Evaluation, Research type
CASP: Critical Appraisal Skills Programme
