# Supplementary material for: Parents’ experiences of caring for a young child with type 1 diabetes: a systematic review and synthesis of qualitative evidence
Source: BMC Pediatr. 2021 Apr 4;21:160. doi: 10.1186/s12887-021-02569-4 (PMC8019496; doi:10.1186/s12887-021-02569-4)
Supplement: Supplementary file 1 — Additional file 1: Table S1. CASP quality appraisal scoresheet [file 12887_2021_2569_MOESM1_ESM.docx]

*Supplementary information*

**Table S1. CASP quality appraisal scoresheet**

| **Study reference** | **Clear statement of aims?** | **Qualitative methodology appropriate?** | **Research design appropriate?** | **Recruitment strategy appropriate?** | **Data collection approach appropriate?** | **Researcher-participant relationship considered?** | **Ethical issues considered?** | **Data analysis sufficiently rigorous?** | **Clear statement of findings?** | **How valuable is this research?** |
| --- | --- | --- | --- | --- | --- | --- | --- | --- | --- | --- |
| Boman et al, 2013 | Y | Y | Y | Y | Y | Y | Y | Y | Y | G |
| Elissa et al, 2017 | Y | Y | Y | Y | Y | CT | Y | Y | Y | G |
| Iversen et al, 2018 | Y | Y | Y | Y | Y | CT | Y | Y | Y | M |
| Khandan et al, 2018 | Y | Y | Y | Y | Y | Y | Y | Y | Y | G |
| Lawton et al, 2015 | Y | Y | Y | Y | Y | Y | Y | CT | Y | G |
| Lindstrom et al, 2017 | Y | Y | Y | Y | Y | Y | Y | Y | Y | G |
| Marshall et al, 2009 | Y | Y | Y | CT | Y | Y | Y | Y | Y | M |
| Patton et al, 2016 | Y | Y | Y | Y | Y | CT | CT | CT | Y | L |
| Perez et al, 2018 | Y | Y | Y | Y | Y | CT | CT | Y | Y | G |
| Rankin et al, 2015 | Y | Y | Y | Y | Y | CT | Y | Y | Y | G |
| Sullivan-Bolyai et al, 2003 | Y | Y | Y | Y | Y | CT | Y | Y | Y | G |
| Sullivan-Bolyai et al, 2004 | Y | Y | Y | Y | Y | CT | CT | Y | Y | G |
| Sullivan-Bolyai et al, 2006 | Y | Y | Y | Y | Y | CT | CT | Y | Y | G |
| Watt, 2017 | Y | Y | Y | Y | Y | Y | Y | Y | Y | M |

*Scoring: Y=Yes; CT=Cannot tell; G=Good; M=Medium; L=Low*
