# Supplementary material for: Parents’ experiences of caring for a young child with type 1 diabetes: a systematic review and synthesis of qualitative evidence
Source: BMC Pediatr. 2021 Apr 4;21:160. doi: 10.1186/s12887-021-02569-4 (PMC8019496; doi:10.1186/s12887-021-02569-4)
Supplement: Supplementary file 2 — Additional file 2: Fig. S1. Exemplar search strategy from Medline database [file 12887_2021_2569_MOESM2_ESM.docx]

*Supplementary information*

**Fig. S1. Exemplar search strategy from Medline database**

1. exp Diabetes Mellitus, Type 1/

2. (IDDM or T1DM or T1D).mp.

3. ("insulin$ depend$" or "insulin?depend$ or insulin-depend$").mp.

4. ("typ? 1 diabet*" or "typ? I diabet*" or "typ?1 diabet$" or "typ?I diabet$" or "auto?immune diabet$").mp.

5. ((juvenile$ or child$) adj2 diabet$).mp.

6. 1 or 2 or 3 or 4 or 5

7. exp Parents/

8. (parent$ or mother$ or father$ or caregiver$ or care$giver$ or mum$ or dad$ or carer$ or guardian$).mp.

9. 7 or 8

10. 6 and 9

11. exp Qualitative Research/

12. (qualitative$ or ethnograph$ or experience$ or interview$ or focus group$ or phenomenol$ or observation$ or perception$ or view$ or (grounded adj theory) or (framework adj analysis) or (thematic adj analysis) or (constant adj comparison)).mp.

13. 11 or 12

14. 10 and 13

15. limit 14 to (english language and yr="2002-Current")
